# The Inverse Relationship between the Microstructural Variability of Amygdala-Prefrontal Pathways and Trait Anxiety Is Moderated by Sex

**DOI:** 10.3389/fnsys.2016.00093

**Published:** 2016-11-16

**Authors:** M. Justin Kim, Annemarie C. Brown, Alison M. Mattek, Samantha J. Chavez, James M. Taylor, Amy L. Palmer, Yu-Chien Wu, Paul J. Whalen

**Affiliations:** ^1^Department of Psychological and Brain Sciences, Dartmouth CollegeHanover, NH, USA; ^2^Division of Epidemiology, The Ohio State University College of Public HealthColumbus, OH, USA; ^3^Department of Radiology and Imaging Sciences, Indiana University School of MedicineIndianapolis, IN, USA

**Keywords:** amygdala, ventral prefrontal cortex, anxiety, white matter, connectivity

## Abstract

Anxiety impacts the quality of everyday life and may facilitate the development of affective disorders, possibly through concurrent alterations in neural circuitry. Findings from multimodal neuroimaging studies suggest that trait-anxious individuals may have a reduced capacity for efficient communication between the amygdala and the ventral prefrontal cortex (vPFC). A diffusion-weighted imaging protocol with 61 directions was used to identify lateral and medial amygdala-vPFC white matter pathways. The structural integrity of both pathways was inversely correlated with self-reported levels of trait anxiety. When this mask from our first dataset was then applied to an independent validation dataset, both pathways again showed a consistent inverse relationship with trait anxiety. Importantly, a moderating effect of sex was found, demonstrating that the observed brain-anxiety relationship was stronger in females. These data reveal a potential neuroanatomical mediator of previously documented functional alterations in amygdala-prefrontal connectivity that is associated with trait anxiety, which might prove informative for future studies of psychopathology.

## Introduction

Altered function of the neural circuitry comprising the amygdala and the prefrontal cortex (PFC) has often been associated with anxiety (LeDoux, 2000; Davis and Whalen, 2001; Bishop, 2007; Hartley and Phelps, 2009; Casey et al., 2010; Milad and Quirk, 2012; Grupe and Nitschke, 2013), and this brain-anxiety relationship has been found in both humans and nonhuman primates (Birn et al., 2014). The utility of this network approach is also supported by developmental studies demonstrating that the amygdala-PFC circuitry is impacted by early life stress (Burghy et al., 2012; Malter Cohen et al., 2013) as well as separation anxiety in children (Gee et al., 2014).

In our previous work, using diffusion magnetic resonance imaging (dMRI)—an imaging method that measures water diffusivity in brain tissue using MRI—we demonstrated that the structural integrity of a white matter pathway between the amygdala and the ventral PFC (vPFC) was inversely correlated with self-reported levels of trait anxiety (Kim and Whalen, 2009; see also Phan et al., 2009). These findings complemented a series of functional MRI studies reporting an inverse relationship between the functional connectivity between the amygdala-vPFC circuitry and anxiety (Pezawas et al., 2005; Kim et al., 2011a; Burghy et al., 2012; Coombs et al., 2014; Bijsterbosch et al., 2015), and offered an anatomical basis for our conceptual framework—namely, that the capacity for efficient communication between the amygdala and the vPFC may be one of the key factors in the regulation and control of anxiety (Kim et al., 2011b).

Notably, a number of dMRI-based analyses of amygdala-vPFC circuitry have not only detected a lateral white matter pathway that includes parts of the uncinate fasciculus—a major fiber tract that interconnects the anterior temporal lobe and the inferior frontal cortex (Ebeling and von Cramon, 1992)—but have also identified a more medial white matter pathway (Johansen-Berg et al., 2008; Kim and Whalen, 2009; Clewett et al., 2014). The separation between these two white matter pathways becomes clearly distinguishable in the proximity of the striatum, as the lateral amygdala-vPFC pathway travels mostly in the external and the extreme capsules, coinciding with the uncinate fasciculus (Bucy and Kluver, 1955), whereas the medial amygdala-vPFC pathway passes through the ventral striatal areas (Fudge et al., 2002). The existence of both medial and lateral pathways connecting the amygdala and the PFC is further supported by tracing studies in nonhuman primates (Ghashghaei et al., 2007; Aggleton et al., 2015).

In light of these neuroanatomical data, our primary objective was to test the possibility that the two amygdala-vPFC pathways are differentially related to anxiety, in order to advance our current understanding of this particular brain-anxiety relationship. To achieve this goal, in an exploratory analysis, we used probabilistic tractography methods to delineate the lateral and medial amygdala-vPFC pathways by separately visualizing white matter tracts that respectively connect the amygdala with the lateral orbitofrontal cortex (lOFC) and the ventromedial PFC (vmPFC)—two distinct vPFC regions known to have dense anatomical connections with the amygdala (Amaral et al., 1992; Ghashghaei et al., 2007; Aggleton et al., 2015)—and tested the relationship between the structural integrity of the two amygdala-vPFC pathways and self-reported levels of trait anxiety. We also tested for a potential moderating effect of sex on this brain-anxiety relationship, based on an animal literature showing that stress-induced changes in the limbic regions of the brain during development are associated with anxiety-like behaviors, especially in females (Weinstock, 2007; Lupien et al., 2009; Bourke et al., 2012), and a recent human functional neuroimaging study reporting a female-specific inverse relationship between the strength of amygdala-vPFC connectivity and self-reported anxiety levels (Burghy et al., 2012). Finally, given that some dMRI studies have argued against our initial finding that the structural integrity of an amygdala-vPFC pathway is inversely correlated with trait anxiety (Montag et al., 2012; Modi et al., 2013; Clewett et al., 2014), there was a need to determine the reproducibility of our original finding using multiple independent datasets with a substantially larger sample size than our original report (Kim and Whalen, 2009) along with updated data analysis procedures described in the “Materials and Methods” Section. Utilizing a 61 direction dMRI protocol that allows for greater angular sampling resolution when resolving crossing fibers, our *a priori* aim was to use these data to define the medial and lateral amygdala-prefrontal pathways in our first dataset, and then to apply this anatomical mask to two additional separate datasets to show the reproducibility of the amygdala-prefrontal structural correlation with reported anxiety.

## Materials and Methods

### Participants

The present report consists of three separate datasets comprising 282 healthy volunteers. Data from 37 individuals were removed from further analyses due to image quality issues, including artifacts, distortions, or excessive head motion (>1.5 mm). Thus, a total of 245 healthy volunteers (121 females; 19.59 ± 1.87 years of age; 241 right-handed) comprising three independent datasets were included in the current study. Categorization of the three datasets was based on the different imaging protocols used to acquire the diffusion-weighted images (see next section for details). Dataset 1 consisted of 71 participants (43 females; 19.49 ± 1.44 years of age; 71 right-handed); Dataset 2 consisted of 120 participants (49 females; 19.72 ± 2.23 years of age; 117 right-handed); Dataset 3 consisted of 54 participants (29 females, 19.44 ± 1.49; years of age; 53 right-handed) recruited from the Dartmouth community. All participants were screened for current or past psychiatric illness using the Structured Clinical Interview for DSM-IV. None of the participants reported being diagnosed with current or past psychiatric disorders (axis I or II). The study received approval and was carried out in accordance with the guidelines set by the Committee for the Protection of Human Subjects at Dartmouth College. Prior to the experiment, written informed consent was acquired from all participants. Self-reported levels of trait anxiety were measured using the State Trait Anxiety Inventory (STAI) Form Y (Spielberger et al., 1988). Handedness was assessed using the Edinburgh Handedness Inventory (Oldfield, 1971). Demographic information of the participants in all three datasets is summarized in Table 1.

**Table 1:** **Demographic characteristics of all participants**.

	Dataset 1 (*n* = 71)		Dataset 2 (*n* = 120)		Dataset 3 (*n* = 54)	
Demographics	Mean (*n*)	SD (%)	Mean (*n*)	SD (%)	Mean (*n*)	SD (%)
Age (years)	19.49	1.44	19.72	2.22	19.44	1.49
Sex (female)	43	60.56	49	40.83	29	53.70
Handedness (right)	71	100	117.00	97.50	53	98.15
Trait anxiety (STAI-T)	34.11	9.17	37.02	10.51	33.02	8.04
Females	34.60	9.14	39.33	9.30	33.00	8.11
Males	33.34	9.33	35.42	11.06	33.04	8.13
Head motion (RMS, mm)	0.43	0.09	0.23	0.07	0.24	0.05

**Fractional Anisotropy**	**Mean**	**SD**	**Mean**	**SD**	**Mean**	**SD**

Amygdala-lOFC
R	0.42	0.01	0.44	0.02	0.40	0.02
L	0.41	0.01	0.43	0.02	0.40	0.02
Amygdala-vmPFC
R	0.42	0.01	0.44	0.02	0.38	0.02
L	0.40	0.01	0.42	0.02	0.35	0.02

### Image Acquisition

Diffusion-weighted images from all 245 participants were obtained at the Dartmouth Brain Imaging Center using the 3.0 Tesla Philips Intera Achieva Scanner (Philips Medical Systems), equipped with a 32-channel head coil (Datasets 1 and 2) and an 8-channel head coil (Dataset 3). For both multichannel receiver coils, sensitivity encoding (SENSE) parallel imaging with a reduction factor of 2 was used to reduce scan time and geometric distortion in the diffusion-weighted images acquired by the fast MRI pulse sequence, single-shot spin-echo echo planar imaging. Separate image acquisition protocols were used for the three datasets. For Dataset 1, diffusion-weighted images were collected with 60 contiguous 2 mm thick axial slices and 61 noncollinear diffusion gradients (echo time (TE) = 85 ms, repetition time (TR) = 4008 ms, *b* value = 1000 s/mm^2^, field of view (FOV) = 224 mm, flip angle = 90°, voxel size = 2 mm × 2 mm × 2 mm). For Dataset 2, diffusion-weighted images were collected with 70 contiguous 2 mm thick axial slices and 32 non-collinear diffusion gradients (TE = 91 ms, TR = 8848 ms, *b* value = 1000 s/mm^2^, FOV = 240 mm, flip angle = 90°, voxel size = 1.875 mm × 1.875 mm × 2 mm). For Dataset 3, diffusion-weighted images were collected with 70 contiguous 2 mm thick axial slices and 32 non-colinear diffusion gradients (TE = 91 ms, TR = 8842 ms, *b* value = 1000 s/mm^2^, FOV = 240 mm, flip angle = 90°, voxel size = 1.875 mm × 1.875 mm × 2 mm). High-resolution anatomical T1-weighted magnetic resonance images (MRI) were scanned using a magnetization-prepared rapid gradient echo sequence (MP-RAGE), with 220 sagittal slices (TE = 3.7 ms, TR = 8.2 ms, FOV = 240 mm, flip angle = 8°, voxel size = 0.9375 mm × 0.9375 mm × 1 mm) from all participants across Datasets 1 and 2. For Dataset 3, an MP-RAGE sequence with 160 sagittal slices was used instead (TE = 4.6 ms, TR = 9.8 ms, FOV = 240 mm, flip angle = 8°, voxel size = 1 mm × 0.9375 mm × 0.9375 mm). The order of data collection was Dataset 3, 2, then 1.

### Data Analysis

#### Behavioral Data

Prior to further statistical analyses, trait anxiety (STAI-T) scores were log transformed in order to account for the non-normal distribution found in the data. The log transformed STAI-T scores were normally distributed in all datasets (Kolmogorov-Smirnov tests, all *p*s > 0.05; further confirmation by visually inspecting the Q-Q plots). Thus, Pearson’s correlation coefficient *r* was used in all three datasets. For all analyses, the effects of age, sex and head motion were controlled using partial correlation, and the statistical threshold for multiple tests was corrected for false discovery rate (FDR; *q* < 0.05; Benjamini and Hochberg, 1995). As a part of a *post hoc* investigation, moderator analyses were performed using the PROCESS macro (Hayes, 2013) in SPSS 21 (IBM Corp., Armonk, NY, USA) to explore the possibility of sex differences in the relationship between the structural integrity of the amygdala-vPFC pathways and trait anxiety, while controlling for age and head motion.

##### Meta-Analysis

Since there were three independent datasets included in the current study, a meta-analytic approach was taken to assess the general relationship between the structural integrity of amygdala-vPFC pathways and trait anxiety. A random-effects model by Hedges and Velvea (1998) was used for the meta-analysis. We used the Pearson correlation coefficient *r*, derived from the partial correlation analyses described above, as an index for effect size. Following the procedure described by Field and Gillett (2010), all correlation coefficients were transformed to Fisher’s *z* scale for meta-analysis, and then converted back to correlation coefficients for reporting. This procedure was applied to the four amygdala-PFC pathways separately.

#### Diffusion-Weighted Imaging Data

All dMRI data were analyzed according to the following four steps: (1) preprocessing using standard procedures using FSL; (2) quantifying head motion; (3) defining regions of interest (amygdala, vPFC) to be used as seed masks for tractography; and (4) performing probabilistic tractography analyses.

##### Preprocessing

Prior to further data processing, all diffusion-weighted images were visually inspected for artifacts, distortions and major head motions to ensure quality. All diffusion-weighted and high-resolution anatomical images were analyzed using the FSL software package and its Diffusion Toolbox (Behrens et al., 2003; Smith et al., 2004). Diffusion-weighted images were preprocessed following standard procedures including brain extraction/skull-stripping, and correcting for eddy current, as well as motion. Head motion was corrected by aligning all diffusion-weighted images to the non-diffusion-weighted (b0) image using affine registration (Jenkinson et al., 2002). To prepare for probabilistic tractography analysis, a dual-fiber model was fitted to the data, which accounts for uncertainties associated with crossing fibers (Behrens et al., 2007). While we did not rotate the b-matrix after eddy/motion correction, given that we exercised a stringent head motion threshold, the potential impact of not rotating the b-matrix would have been minimized. We do note that correcting the b-matrix after eddy/motion correction may further reduce directional bias due to motion (Leemans and Jones, 2009).

##### Head Motion

Given the possibility that head motion could drive spurious outcomes by altering diffusivity- or anisotropy-related measures derived from diffusion-weighted images (Yendiki et al., 2013), we sought to evaluate the degree of head motion for each participant and test for its potential correlation with trait anxiety. To achieve this, volume-by-volume head motion was quantified by calculating the root mean square (RMS) deviation of the six motion parameters (three translation and three rotation components), which were derived from the affine registration between each volume to the b0 image, for each pair of consecutive diffusion-weighted brain volumes (Jenkinson et al., 2002). The resulting volume-by-volume RMS deviation values were averaged across all images, yielding a summary statistic of head motion for each participant.

##### Region-of-Interest Definition

**Figure 1:**
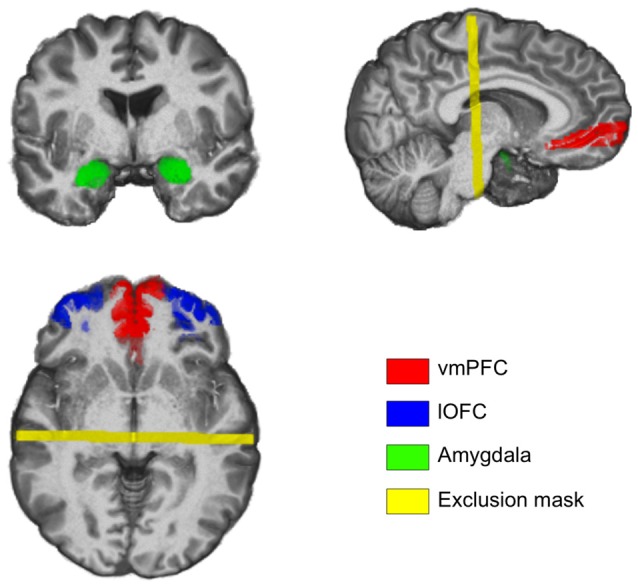
**Coronal, sagittal and axial slices of the brain depicting the regions of interest (ROIs) used to define amygdala-ventral prefrontal cortex (vPFC) pathways.** Images are taken from a representative subject’s native structural space.

Given the hypothesis of the current study, our regions-of-interest (ROIs) were focused on the amygdala and the ventral PFC areas. In order to tailor the ROIs to each participant, subject-specific ROIs were created using their own high-resolution anatomical images. The amygdala ROI was defined using an automated subcortical segmentation tool that is implemented in FSL (FIRST; Patenaude et al., 2011). To ensure the quality of the segmentation, the resulting amygdala ROIs were visually inspected for gross errors on a subject-by-subject basis and confirmed that the segmented amygdala did not contain significant portions of the hippocampus. However, it is important to note that the automated segmentation of the amygdala may have resulted in the inclusion of a small amount of the neighboring tissue, mainly the hippocampus. Despite this possibility, given that our focus was on the white matter tracts that run between the amygdala and the PFC while excluding anything that goes posterior from the amygdala, this is expected to have minimal impact on our overall findings. For the ventral PFC, we selected two ROIs—the ventromedial wall of the PFC, and the ventrolateral surface of the orbitofrontal cortex (OFC). We defined these ROIs using the Automated Anatomical Labeling atlas (labeled *Frontal_Med_Orb* and *Frontal_Mid_Orb*, respectively), which are in standard Montreal Neurological Institute (MNI) space (Maldjian et al., 2003). These ROIs were subsequently warped into each participant’s anatomical space using nonlinear transformation. Then, for each participant, the gray matter tissue was segmented from their own anatomical images using the automated segmentation tool in FSL (FAST; Zhang et al., 2001). Adapting the procedures described by Clewett et al. (2014), the gray matter images were thresholded to ensure that voxels with at least 35% probability of being gray matter were selected. Finally, these thresholded gray matter images were masked with the warped ventral PFC ROIs. This procedure resulted in the creation of subject-specific ROIs of the two ventral PFC ROIs that only included gray matter tissue in each participant’s anatomical space. We will refer to these two ROIs—the ventromedial wall of the PFC and the ventrolateral surface of the OFC—as the vmPFC and the lOFC, respectively (Figure 1). The vmPFC ROIs include Brodmann areas 10 and 11, whereas the lOFC ROIs include Brodmann areas 11, 46 and 47.

##### Probabilistic Tractography Analysis

Using subject-specific ROIs as seed regions, separate probabilistic tractography analyses were performed on Dataset 1 (*n* = 71) to identify white matter tracts that run between the amygdala and the vmPFC/lOFC on a subject-by-subject basis. We used 5000 probabilistic tract streamlines for each voxel in the seed regions. Two sets of probabilistic tractography analyses were carried out for each pair of ROIs, one for each direction—that is, if the first analysis would use the amygdala as the seed region and the vmPFC as the target region, then the second analysis would have the vmPFC as the seed region and the amygdala as the target region. Only the white matter tracts that survived both procedures were used in subsequent group-level analyses to minimize Type I error. An exclusion mask was imposed at *y* = −20 in order to exclude tracts that runs posterior to the hippocampus. This resulted in a total of four white matter tracts per subject: the amygdala-vmPFC (medial) pathway and the amygdala-lOFC (lateral) pathway, for each hemisphere. These white matter tracts, which were stored in each participant’s own native diffusion space, were spatially normalized into standard MNI space (2 mm × 2 mm × 2 mm) using a two-step registration process in order to enable group-level analyses. To achieve this, for all datasets, diffusion-weighted images were first registered to the high-resolution anatomical images, which were then warped into standard MNI space using nonlinear registration (FNIRT). Transformation matrices derived from these two steps were concatenated, resulting in a diffusion-to-standard transformation matrix that was applied to each of the four white matter tracts. To create group-level tracts, these four white matter tracts were binarized and then overlaid onto a single map, and subsequently thresholded to 50% probability across subjects—an approach that was previously shown to be useful in examining individual differences (Chavez and Heatherton, 2015). This allowed for the resulting group-level white matter tracts to only include voxels with at least 50% of the subjects showing agreement (Figure 1). The structural integrity of the white matter tracts was defined as the average fractional anisotropy (FA) values across all voxels included in the group-level tracts. For each subject, average FA values from each white matter tract (thresholded to ensure that voxels with FA <0.2 were excluded) were extracted for further correlation analyses with trait anxiety. To extract average FA values, first the four white matter tracts were transformed back to each subject’s own native diffusion space by applying the inverse of the diffusion-to-standard transformation matrix; then, average FA values were calculated from the original diffusion-weighted images. The outcomes of each preprocessing step were visually inspected to ensure quality. We note here that Datasets 2 and 3 did not yield sufficient white matter tracts using the same parameters for the probabilistic tractography analyses. This discrepancy in tractography outcomes may be attributable to the substantial differences in the number of diffusion gradients used to acquire the images (61 vs. 32), with higher angular sampling resolution allowing for more sensitive detection of white matter tracts. In light of this, instead of lowering the threshold of the tracts defined from Datasets 2 or 3, we chose to apply the group-level white matter tracts defined from Dataset 1 to Datasets 2 and 3 for further correlation analyses—this procedure would serve as a test to assess the generalizability of the findings from Dataset 1 using multiple independent datasets. While the caveat of this approach is a potential Type II error, it serves to reduce Type I error. Finally, to test the possibility that certain subregions of amygdala-vPFC pathways may be selectively correlated with trait anxiety, a supplementary voxelwise analysis was performed (Supplementary Materials and Methods).

## Results

### Tractography Results

**Figure 2:**
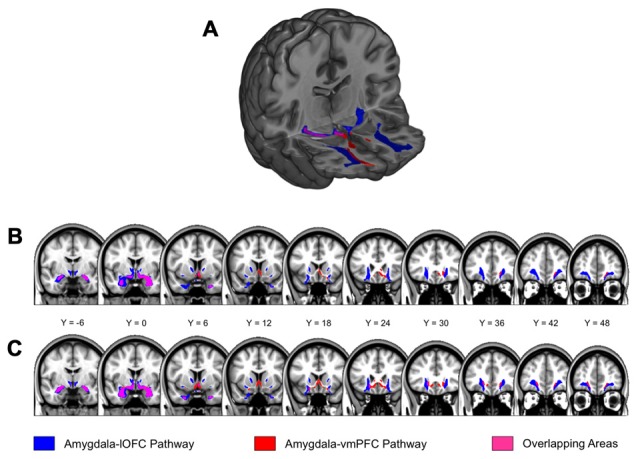
**Amygdala-lateral orbitofrontal cortex (lOFC) and amygdala-ventromedial PFC (vmPFC) pathways. (A)** Results from probabilistic tractography illustrating the amygdala-lOFC pathway (blue), the amygdala-vmPFC pathway (red), and the overlapping pathway (purple). Tracts are displayed at a group threshold of 50%. **(B)** Pathways depicted on coronal slices of the brain, defined at a group threshold of 50%. **(C)** A lenient group threshold of 30% was used for the left amygdala-vmPFC pathway. The main findings did not change when this threshold was applied to the left amygdala-vmPFC pathway.

Probabilistic tractography analyses of the diffusion images (Dataset 1) revealed two white matter tracts within each hemisphere—one connecting the amygdala with the vmPFC, and the other connecting the amygdala with the lOFC (Figure 2). The two white matter tracts overlapped in the ventral basal forebrain in close proximity to the amygdala, which was expected due to the use of a common amygdala anatomical seed ROI. A separation between these two white matter tracts gradually became noticeable near the striatum: the amygdala-vmPFC pathway comprised white matter passing through the ventral striatum into the PFC while the amygdala-lOFC pathway included parts of the external capsule, and to a smaller extent, the anterior limb of the internal capsule that extended into the PFC. These two white matter tracts were separable all the way to their termination within the vmPFC and the lOFC, respectively.

At the current group-level threshold, we observed a substantial left-right asymmetry in the size of the white matter tracts in the amygdala-vmPFC pathway, with the volume of the left tract being approximately 25% of its right counterpart (left: 1704 mm^3^ vs. right: 6640 mm^3^). In contrast, the volume of the amygdala-lOFC pathway was comparable across hemispheres (left: 13904 mm^3^ vs. right: 9672 mm^3^). In an attempt to mitigate the potential influence of these volumetric differences across hemispheres on the brain-anxiety relationship, we ran a series of additional analyses for the left amygdala-vmPFC pathway using a relatively lenient group threshold of 30%, which yielded a size for the left that was comparable with the right pathway (left: 7272 mm^3^ vs. right: 6640 mm^3^; Figure 2). We note here that the main findings were not affected when a group threshold of 30% was used for the left amygdala-vmPFC pathway.

### Head Motion and Trait Anxiety

All participants included in the subsequent analyses (see “Participants” Section) had an acceptable degree of head motion (average range: 0.13–0.75 mm). No significant correlation between head motion and trait anxiety was observed in Dataset 1 (*r* = 0.099, *p* = 0.413), Dataset 2 (*r* = −0.032, *p* = 0.725) or Dataset 3 (*r* = −0.013, *p* = 0.926). There were no significant correlations between head motion and average FA values of each white matter tract investigated in the current study.

### Amygdala-vPFC Pathways Strength and Trait Anxiety

**Figure 3:**
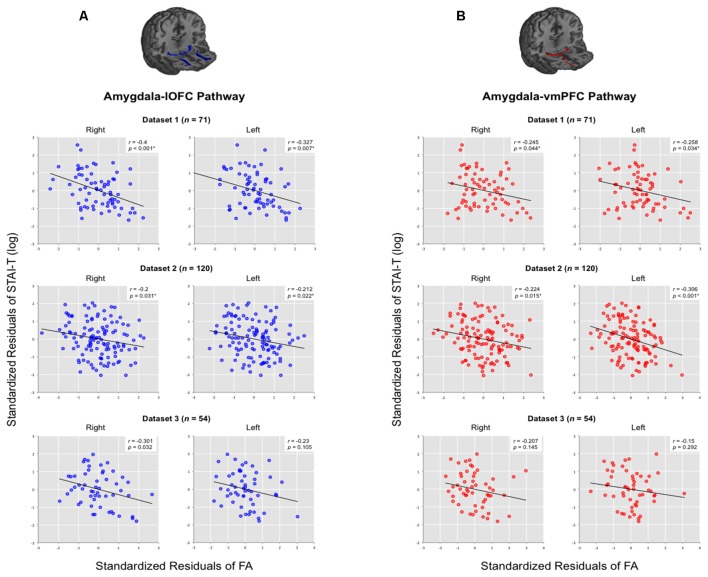
**Correlations between trait anxiety with the structural integrity of amygdala-vPFC pathways.** Results are organized by datasets, separately for **(A)** amygdala-lOFC pathways and **(B)** amygdala-vmPFC pathways. Average fractional anisotropy (FA) values were extracted for each pathway and plotted against trait anxiety, while removing the effects of age, sex and head motion. *q < 0.05.

In Dataset 1, the structural integrity of the amygdala-lOFC white matter tracts, as indexed by average FA, was inversely correlated with STAI-T scores (after controlling for the effects of age, sex and head motion; Figure 3). This significant effect was observed within the right (*r* = −0.4, *p* < 0.001) and left (*r* = −0.327, *p* = 0.007; both *q*s < 0.05) hemispheres (Figure 2). We then applied this tract-specific mask from Dataset 1 to the other two data sets, and also found a significant relationship between the FA values and STAI-T scores, which was observed bilaterally in Dataset 2 (right, *r* = −0.2, *p* = 0.031; left, *r* = −0.212, *p* = 0.022; both *q*s < 0.05), and a nonsignificant trend in Dataset 3 (right, *r* = −0.301, *p* = 0.032; left, *r* = −0.23, *p* = 0.105). Meta-analysis identified significant negative correlations for the right lOFC pathway (mean *r* = −0.283, 95% CI [−0.4, −0.158], *z* = 4.316, *p* < 0.001) and left lOFC pathway (mean *r* = −0.25, 95% CI [−0.365, −0.127], *z* = 3.92, *p* < 0.001). Thus, the integrity of the right lOFC pathway showed a consistent inverse relationship with trait anxiety, with weaker connectivity associated with higher reported anxiety.

Analysis of the vmPFC pathways showed a similar relationship between pathway integrity and trait anxiety (Figure 3). We observed a significant effect within Dataset 1 (right: *r* = −0.245, *p* = 0.044; left: *r* = −0.258, *p* = 0.034; both *q*s < 0.05). When this tract-specific mask was applied to the other two data sets, we also found a significant inverse relationship between tract integrity and anxiety in Dataset 2 (right, *r* = −0.224, *p* = 0.015; left, *r* = −0.306, *p* < 0.001; both *q*s < 0.05). A nonsignificant trend was found in Dataset 3 (right, *r* = −0.207, *p* = 0.145; left, *r* = −0.15, *p* = 0.292). Meta-analysis showed significant negative correlations for the right vmPFC pathway (mean *r* = −0.226, 95% CI [−0.343, −0.102], *z* = 3.54, *p* < 0.001) and left vmPFC pathway (mean *r* = −0.259, 95% CI [−0.374, −0.137], *z* = 4.078, *p* < 0.001).

### Sex Differences in the Present Structural Connectivity-Anxiety Correlation

**Figure 4:**
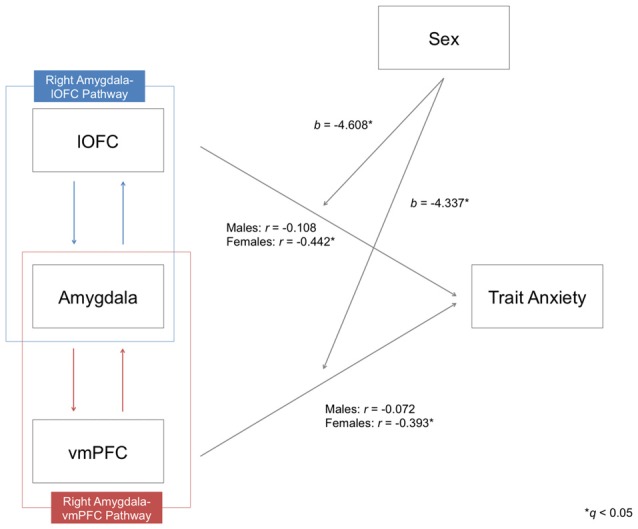
**Moderating effects of sex on the relationship between the structural integrity of the right amygdala-lOFC/vmPFC pathways and trait anxiety.** Partial correlation coefficients (*r*) were calculated by controlling the effects of age and head motion. Moderating effects of sex are marked with unstandardized coefficients (*b*). *q < 0.05.

Given the previous evidence showing that the relationship between prefrontal-amygdala function and anxiety can be stronger in females (Burghy et al., 2012), we conducted a moderator analysis to determine if any such effect existed within the present data. For this analysis, we combined the three datasets to attain maximal statistical power (124 males vs. 121 females). Prior to merging the data, individual FA values of each pathway were mean-centered with respect to its group average FA for each dataset, in order to remove any potential between group effects across datasets. A significant moderating effect of sex was identified only for the pathways in the right hemisphere in predicting trait anxiety, after controlling for the effects of age and head motion (right amygdala-lOFC pathway (*b* = −4.608, *t* = −2.413, *p* = 0.017)), right amygdala-vmPFC pathway (*b* = −4.337, *t* = −2.361, *p* = 0.019; both *q*s < 0.05; see Figure 4). This effect was not found for left hemisphere pathways.

**Figure 5:**
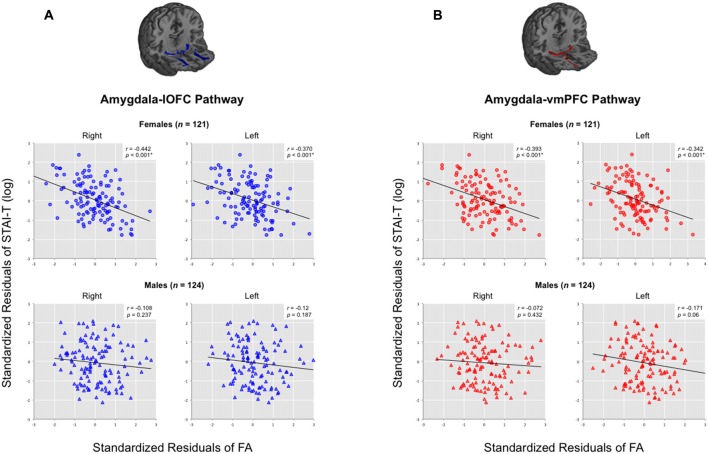
**Correlations between trait anxiety with the structural integrity of amygdala-vPFC pathways across all datasets, organized by sex.** Scatterplots are presented separately for **(A)** amygdala-lOFC pathways and **(B)** amygdala-vmPFC pathways. Average FA values were extracted for each pathway and plotted against trait anxiety, while removing the effects of age and head motion. *q < 0.05.

Upon further inspection, we confirmed that there was a clear distinction between the two sexes: while males did not exhibit a significant correlation between tract integrity and trait anxiety scores in any pathway (all *p*s > 0.05), females showed significant inverse correlations for all four amygdala-vPFC pathways (right amygdala-lOFC: *r* = −0.442, *p* < 0.000001; right amygdala-vmPFC: *r* = −0.393, *p* = 0.00001; left amygdala-lOFC: *r* = −0.37, *p* = 0.000035; left amygdala-vmPFC: *r* = −0.342, *p* = 0.00014; see Figure 5 and Supplementary Table 1). The observed effect of sex was not attributable to group differences in the variance of the measures (e.g., males did not have limited range/skewed distribution in their anxiety scores or white matter FA values compared to females; Levene’s test, all *p*s > 0.05).

Given the general tendency for females to show strong inverse correlations between trait anxiety and the structural integrity of both amygdala-lOFC/vmPFC pathways, there was a need to rule out the possibility that trait-anxious females might be simply displaying lower FA values in limbic white matter tracts in general. To test this idea, we selected the cingulum bundle—another prominent white matter tract in the limbic system—as a control pathway. The bilateral cingulum bundle was defined using the Johns Hopkins University DTI-based white matter atlas provided with the FSL software package (Wakana et al., 2007), and its average FA was extracted for each participant. After applying the same aforementioned partial correlation procedure, we found that the structural integrity of the bilateral cingulum bundle was not significantly correlated with trait anxiety in either sex subgroups (all *q*s > 0.05), effectively demonstrating that the observed brain-anxiety relationship is not due to a general decrease in the structural integrity of limbic system connectivity. In addition, the structural integrity of the bilateral cingulum bundle was not significantly correlated with trait anxiety when both sexes were included in the analysis (all *q*s > 0.05).

## Discussion

Here we further delineate the relationship between the structural integrity of the white matter tracts between the amygdala and the vPFC, and individual differences in self-reported levels of trait anxiety. In addition to supporting the findings of our previous report (Kim and Whalen, 2009), the present study used separate subject-specific ROI and probabilistic tractography methods on diffusion images collected across multiple independent datasets to delineate two separable pathways and tested their relationship with anxiety. Specifically, both pathways showed an inverse relationship with trait anxiety, where lower tract integrity predicted higher reported anxiety levels. A separate moderator analysis revealed a modulatory role of sex, such that females, compared to males, showed a significantly stronger brain-behavior relationship in the right hemisphere. Given that our main findings were derived while controlling for the effects attributable to sex differences, this suggests that while the inverse correlation between amygdala-vPFC pathway strength and anxiety is stronger in females, this brain-anxiety relationship could be observed in the overall study sample.

Our data complement and expand previous findings from other groups that utilized dMRI to show an inverse relationship between frontotemporal white matter strength and trait anxiety/anxiety-related constructs (e.g., harm avoidance, neuroticism) in nonclinical populations (Kazlouski et al., 2011; Motzkin et al., 2011; Westlye et al., 2011; Taddei et al., 2012; Bjørnebekk et al., 2013; Mettler et al., 2013; Eden et al., 2015; Greening and Mitchell, 2015). Collectively, these findings support the notion that stronger connectivity between the amygdala and the vPFC reflects the capacity for efficient crosstalk between these brain regions that ultimately leads to beneficial outcomes in terms of anxiety (Kim et al., 2011b). Given accumulating findings demonstrating impaired structural integrity of the amygdala-vPFC pathway in pathological anxiety disorders such as generalized anxiety disorder (Hettema et al., 2012; Tromp et al., 2012; Cha et al., 2014; Liao et al., 2014) and social anxiety disorder (Phan et al., 2009; Baur et al., 2011), findings from the current study may prove useful in guiding future clinical investigations.

What we add to the existing knowledge is a modulatory role of sex where females exhibited strong inverse correlations in most of the investigated white matter pathways, whereas males displayed a weaker brain-anxiety relationship. The current data are consistent with a recent finding that highlights a female-specific inverse relationship between amygdala-vPFC resting state functional connectivity and self-reported anxiety symptoms (Burghy et al., 2012). Further, a number of previous studies showing an inverse relationship between the structural integrity of an amygdala-vPFC pathway and trait anxiety consisted of dMRI data derived entirely from females (Kazlouski et al., 2011; Mettler et al., 2013; Eden et al., 2015). Finally, an additional study showed a female-specific positive correlation between the structural integrity of the uncinate fasciculus and self-reported use of reappraisal strategy during emotion regulation (Zuurbier et al., 2013). While a psychosocial explanation of the observed sex differences is possible (e.g., males may be less accurate than females in evaluating and reporting their own levels of trait anxiety, and some young males may be especially concerned about impression management and would conceal their actual levels of anxiety; Egloff and Schmukle, 2004), findings from nonhuman primates and other animals suggest that at least some dispositional differences can be explained by sex (Weinstock, 2007; Lupien et al., 2009; Bourke et al., 2012). That being said, it is important to point out that the current results were observed primarily from young adults. Chronological age, and its interaction with sex, is likely to play a critical role in the brain-anxiety relationship reported here, as the developmental trajectory of the uncinate fasciculus in humans suggests that this tract fully matures into the twenties (Lebel et al., 2008).

Findings from the animal literature could offer further insight into how our findings might relate to an underlying neural mechanism of anxiety. For example, it is known from anatomical studies of nonhuman primates using anterograde and/or retrograde tracers that the amygdala has widespread reciprocal interconnections between the PFC including both the vmPFC and lOFC (Amaral et al., 1992; Ghashghaei et al., 2007; Aggleton et al., 2015). Specifically, the OFC sends excitatory projections that directly modulate the activity of the intercalated cell masses. Importantly, excitation of the intercalated cell masses inhibits some amygdala output at the level of the central nucleus of the amygdala, which in turn disables its tonic suppression of the hypothalamus (Barbas and Zikopoulos, 2006; Ray and Zald, 2012). We can speculate then that weakened structural connectivity of the amygdala-lOFC pathway could reflect diminished excitatory input from the OFC to the central nucleus of the amygdala, which would produce altered autonomic responses as a consequence of the disinhibited hypothalamus—such altered responses could contribute to elevated levels of trait anxiety.

Some caveats of the current study should be addressed. First, the brain-anxiety relationship was less clear for Dataset 3, relative to Datasets 1 and 2. The fact that brain data in Dataset 3 were collected using an older 8-channel head coil may have contributed to this discrepancy, as it is suggested that a higher number of channels in a head coil (e.g., 32-channel; Datasets 1 and 2) generally improves dMRI image quality (Parikh et al., 2011). Still, we note that the results from Dataset 3 showed a significant (at uncorrected *p* < 0.05) inverse correlation between trait anxiety and the right amygdala-lOFC pathway, and other pathways also exhibited a similar trend. Second, our study sample was limited to young adults, with the vast majority of the participants being either in their late teens or early twenties. Thus, the brain-anxiety relationship illustrated here should be tested in other age groups with adequate sample sizes. Third, the findings described in the present study are correlational in nature, thus we are cautious to not infer any form of causal relationship between the structural integrity of white matter tracts and anxiety. Given that demonstrating causal relationships is key to understanding the neural mechanisms of anxiety disorders and their treatment, adopting methodological approaches such as imaging genetics (Hariri et al., 2006) or long-term longitudinal study designs could reveal the causal nature of this brain-anxiety relationship. Fourth, we suggest caution in interpreting our data in light of the findings from anatomical and physiological studies of animals, as human tractography results could easily include multiple white matter fiber tracts not directly related to the aforementioned pathways due to the limited resolution of dMRI methods. Fifth, while the term “trait anxiety” is generally used to represent the STAI-T total score, it has been suggested that what the STAI-T measures is better described as negative affect (Grupe and Nitschke, 2013), which includes components of both anxiety and depression (Bados et al., 2010). In other words, the STAI-T, despite its namesake, may be indexing more than just trait anxiety. However, in spite of its apparent lack of specificity, the usefulness of the STAI-T lies within its sensitivity in predicting the risk for psychopathology of anxiety disorders (Grupe and Nitschke, 2013). Future studies would benefit from delineating anxiety and depression in the context of amygdala-vPFC circuitry. Sixth, tractography methods alone cannot identify whether specific subregions of the amygdala-vPFC pathways show a particularly strong inverse correlation with trait anxiety (in an attempt to address this, a supplementary voxelwise analysis was performed on the FA images; see Supplementary Figure 1 for details). Finally, it is worth noting that there are publicly available large-scale databases that will allow for the study of larger sample sizes than the current study. These databases such as the Human Connectome Project (Van Essen et al., 2013) and the Brain Genomics Superstruct Project (Holmes et al., 2015) provide structural and functional brain images as well as behavioral data. As more data accumulate over time, so will the utility of these databases, which will not only allow further exploration of the neural basis of anxiety, but also offer a particularly useful means to investigate various brain-behavior relationships.

## Conclusion

To summarize, our data showed that stronger structural connectivity between the amygdala and the ventral PFC was associated with lower levels of trait anxiety, and this brain-anxiety relationship was consistently demonstrated in multiple independent datasets, especially in females. As the underlying anatomical connections are key to efficient crosstalk between multiple brain regions, the current findings offer a possible candidate for a neuroanatomical mediator of functional alterations in the amygdala and the PFC in trait-anxious individuals, which could serve as a link to the future onset of affective disorders.

## Author Contributions

MJK and PJW designed the study, interpreted the findings and wrote the manuscript. MJK analyzed the data. MJK, ACB, AMM, SJC, JMT, ALP collected the data and critically reviewed the manuscript. YCW interpreted the findings and critically reviewed the manuscript. All authors discussed the results, reviewed and edited the manuscript, and approved the final version for submission.

## Funding

This work was supported by the National Institute of Mental Health grants (R01 MH080716 to PJW and F31 MH090672 to MJK).

## Conflict of Interest Statement

The authors declare that the research was conducted in the absence of any commercial or financial relationships that could be construed as a potential conflict of interest. The reviewer DWG and handling Editor declared their shared affiliation, and the handling Editor states that the process nevertheless met the standards of a fair and objective review.
