# Supplementary material for: The Inverse Relationship between the Microstructural Variability of Amygdala-Prefrontal Pathways and Trait Anxiety Is Moderated by Sex
Source: Front Syst Neurosci. 2016 Nov 16;10:93. doi: 10.3389/fnsys.2016.00093 (PMC5110520; doi:10.3389/fnsys.2016.00093)
Supplement: Supplementary file 1 [file Presentation_1.pdf]

Supplementary Material

The inverse relationship between the microstructural variability of amygdala-prefrontal pathways and trait anxiety is moderated by sex

M. Justin Kim*, Annemarie C. Brown, Alison M. Mattek, Samantha J. Chavez, James M. Taylor, Amy L. Palmer, Yu-Chien Wu, Paul J. Whalen

*** Correspondence:** M. Justin Kim: justin.m.kim@dartmouth.edu

Supplementary Materials and Methods

Voxelwise analysis on the fractional anisotropy (FA) images was completed using FSL's Diffusion Toolbox (Behrens et al., 2003; Smith et al., 2004) and SPM8 (Wellcome Department of Imaging Neuroscience, London, UK), following the steps similar to the procedure described by Zuurbier and colleagues (2013). First, FA images derived from the preprocessing step in the main analysis were nonlinearly registered (FNIRT) to standard Montreal Neurological Institute space using the FMRIB58_FA template (1 × 1 × 1 mm). Following registration, all images were smoothed using a Gaussian kernel of 6-mm full-width at half maximum.

A group-level general linear model was constructed in order to perform voxelwise regression analysis on the preprocessed FA images. This model included log-transformed trait anxiety scores as the independent variable. Age, sex, head motion, as well as dummy variables for the three datasets were included as covariates of no interest. FA images from all datasets were entered for this analysis. Given our *a priori* hypothesis, bilateral uncinate fasciculi (UF), which were adapted from the Johns Hopkins University DTI-based white matter atlas (Wakana et al., 2007; Zhang et al., 2010), was used as regions of interest (ROI). A voxelwise significance threshold of $p < 0.05$, family-wise error (FWE) corrected for multiple comparisons, was imposed on these ROIs. Voxels clusters with at least 5 contiguous voxels surviving this threshold are reported. In addition, sex differences in the relationship between the mean FA values of the UF and trait anxiety were also examined.

Supplementary Results

Extending the findings from the main analysis, significant negative correlations between trait anxiety and FA values were observed in two voxel clusters in the left UF (Cluster 1: MNI -38, 0, -5, $t = 5.33$, FWE-corrected $p < 0.001$, $k = 89$ voxels; Cluster 2: MNI -22, 29, -9, $t = 4.76$, FWE-corrected $p < 0.001$, $k = 105$ voxels) and one voxel cluster in the right UF (MNI: 35, 14, -8, $t = 4.25$, FWE-corrected $p < 0.001$, $k = 23$ voxels). These clusters were located proximal to striatum/insula and the orbitofrontal cortex (**Supplementary Figure 1**). No voxels displayed a significant positive correlation between trait anxiety and FA values. Taken together, findings from the voxelwise analysis indicate specific subregions of the UF show a particularly strong inverse relationship with trait anxiety.

When mean FA values of the UF was extracted and submitted to a moderator analysis described in the main analysis, the moderating effects of sex was not significant (Right UF: $b = -2.907$, $t = -1.096$, $p = 0.274$; Left UF: $b = -2.919$, $t = -1.225$, $p = 0.222$), although the correlations were significant for females (Right UF: $r = -0.314$, $p < 0.001$; Left UF: $r = -0.33$, $p < 0.001$) and not significant for males (Right UF: $r = -0.16$, $p = 0.078$; Left UF: $r = -0.168$, $p = 0.064$**)**. These data collectively suggest that the moderating effect of gender can be more clearly observed when considering certain portions of the UF.

Supplementary References

Behrens, T. E., Woolrich, M. W., Jenkinson, M., Johansen-Berg, H., Nunes, R. G., Clare, S., et al. (2003). Characterization and propagation of uncertainty in diffusion-weighted MR imaging. *Magn. Reson Med.* 50, 1077-1088.

Smith, S. M., Jenkinson, M., Woolrich, M. W., Beckmann, C. F., Behrens, T. E., Johansen-Berg, H., et al. (2004). Advances in functional and structural MR image analysis and implementation as FSL. *Neuroimage* 23 Suppl 1, S208-219.

Wakana, S., Caprihan, A., Pazenboeck, M. M., Fallon, J. H., Perry, M., Gollub, R. L., et al. (2007). Reproducibility of quantitative tractography methods applied to cerebral white matter. *Neuroimage* 36, 630-644.

Zhang, Y., Zhang, J., Oishi, K., Faria, A. V., Jiang, H., Li, X., et al. (2010). Atlas-guided tract reconstruction for automated and comprehensive examination of the white matter anatomy. *Neuroimage*, 52, 1289–1301

Zuurbier, L. A., Nikolova, Y. S., Ahs, F. & Hariri, A. R. (2013). Uncinate fasciculus fractional anisotropy correlates with typical use of reappraisal in women but not men. *Emotion* 13, 385-390.

Supplementary Figures

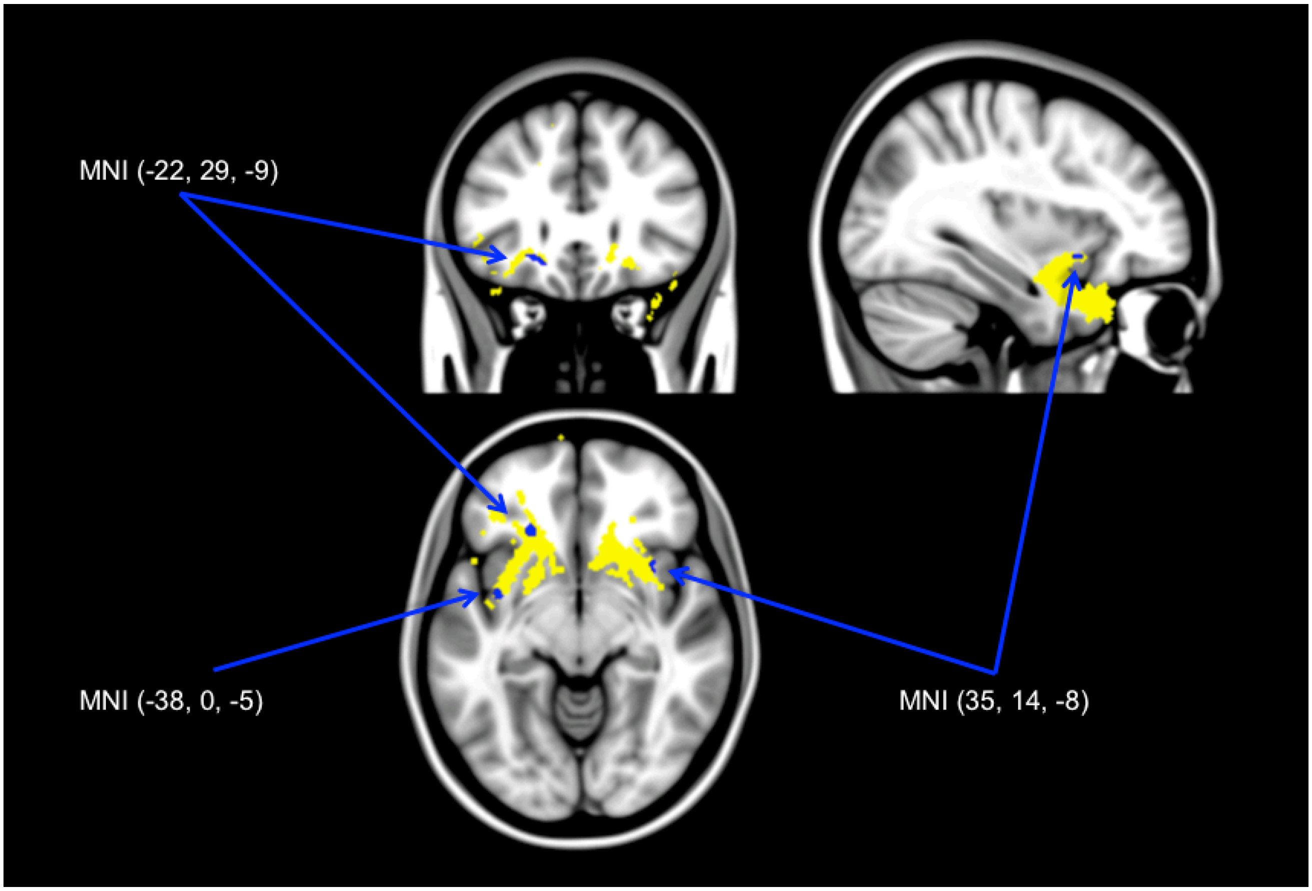

Supplementary Figure 1. Uncinate fasciculus (yellow) and its subregions (blue) showing significant negative correlations between fractional anisotropy values and trait anxiety, while controlling for the effects of age, sex, and head motion (FWE-corrected $p < 0.05$).

Supplementary Tables

Supplementary Table 1. Partial correlation coefficients between the structural integrity of each pathway and trait anxiety organized by sex (controlling for age and head motion) for each dataset.

**Dataset 1**		Male ($n = 28$)		Female ($n = 43$)	
Pathways		*r*	*p*	*r*	*p*
Amygdala-lOFC	R	-0.394	0.046	-0.438	*0.004
	L	-0.105	0.611	-0.494	*0.001
Amygdala-vmPFC	R	-0.169	0.408	-0.291	0.065
	L	0.063	0.760	-0.383	*0.013
**Dataset 2**		Male ($n = 71$)		Female ($n = 49$)	
Pathways		*r*	*p*	*r*	*p*
Amygdala-lOFC	R	-0.042	0.731	-0.494	§*0.000417
	L	-0.119	0.329	-0.395	*0.006065
Amygdala-vmPFC	R	-0.021	0.864	-0.589	§*0.000013
	L	-0.207	0.088	-0.471	*0.000833
**Dataset 3**		Male ($n = 25$)		Female ($n = 29$)	
Pathways		*r*	*p*	*r*	*p*
Amygdala-lOFC	R	-0.352	0.099	-0.267	0.179
	L	-0.257	0.237	-0.227	0.255
Amygdala-vmPFC	R	-0.304	0.159	-0.111	0.582
	L	-0.183	0.403	-0.108	0.593

*Correlation coefficients significant at FDR-corrected $q < 0.05$

§Significant moderating effects of sex at $p < 0.05$ (R amygdala-lOFC pathway: $b = -5.207$, $t = -1.992$, $p = 0.0487$; R amygdala-vmPFC pathway: $b = -5.845$, $t = -2.524$, $p = 0.013$).
